# Conservation Management of the Endangered Asiatic Lions in Gujarat, India, Using GPS Satellite Telemetry

**DOI:** 10.3390/ani13010125

**Published:** 2022-12-28

**Authors:** Mohan Ram, Aradhana Sahu, Nityanand Srivastava, Lahar Jhala, Yashpal Zala, Meena Venkataraman

**Affiliations:** 1 Wildlife Division, Sasan-Gir, Junagadh 362135, GJ, India; 2 Wildlife Circle, Junagadh 362001, GJ, India; 3 Principal Chief Conservator of Forests (Wildlife) & Chief Wildlife Warden, Gandhinagar 382010, GJ, India; 4 Carnivore Conservation and Research, Mumbai 400601, MH, India

**Keywords:** Asiatic lion, dispersal, geofence, GPS cluster, GPS satellite radio-telemetry, immobility

## Abstract

**Simple Summary:**

Asiatic lions are found in the Gir Protected Areas and surrounding multi-use land matrices in Saurashtra, Gujarat. Scientifically monitoring their movement and activity is vital for their conservation management. We used GPS radio-collars to study the movement and activity of 19 individual lions. This pilot study’s results revealed how technology-driven scientific monitoring can help in conserving endangered species. From the conservation management perspective, we discussed the use of virtual geofence functions and alert generation to reduce the chances of endangered species mortality on linear infrastructures. We also presented a pilot case study on how GPS radio-telemetry helped in securing the safety of dispersing sub-adult coalition males extensively moving in a multi-use land matrix. This study may help develop baseline ecological information for impact assessment studies and predictive models for lion ecological requirements, habitat improvement and proactive and retrofitting mitigation measures.

**Abstract:**

Endangered Asiatic lions (*Panthera leo persica*) are found in the Asiatic Lion Landscape, Gujarat, which includes protected areas and a multi-use land matrix. Therefore, monitoring lions’ space-use and spatio-temporal location is vital for managing various facets of human−lion interaction. Our study demonstrates how this was achieved by tracking lions using GPS radio-collars, triggering prompt action via an efficient communication network across political and forest administrative boundaries. We monitored the movement of 19 individual lions for 436.5 ± 32 days and also derived the mean daily activity from three-axis accelerometer-based activity-sensing feature of a radio-collar. We also monitored geofence breaches. We proposed that immobility and movement are two aspects that generate management action on the ground. While the movement is related to ranging and dispersal, immobility is a situation related to either the animal’s feeding or its health status. From a management standpoint, we discussed the efficacy of the virtual geofence in preventing accidents when lions moved and also presented the advantages of being able to track dispersal through a case study of sub-adult lions. To strengthen our response to lion immobility, we developed a predictive model that specifically highlights an individual lion’s health status and makes the alert response more precise. In conclusion, we critically reviewed the capabilities provided by GPS telemetry and provide protocols that help in the conservation management of lions and that will also have a wider application.

## 1. Introduction

Human-wildlife interactions that were earlier reported at protected area interfaces are increasingly being reported even in distant cities, as animals foray farther away from protected areas [1,2]. Monitoring animal movement, particularly endangered species, outside protected areas in multi-use landscapes is a great challenge, with possibilities of retaliatory killing, poaching, hunting and accident-related mortality [3,4]. As species traverse rural and urban settings, navigating across a matrix of land uses and continuously monitoring their health and status is a difficult feat to accomplish without a good understanding of the concerned species’ ecology [5]. Additionally, conservation managers have to mitigate costs for people caused by wildlife and reconcile damage to life, property, and livelihoods in order to gain support for and safeguard wild species’ survival in the multi-use land matrix [6,7]. Real-time monitoring of wildlife movement through the Global Positioning System (GPS) telemetry, further strengthened by improved communication networks, is proving to be a great aid for coping with these novel conservation challenges [8]. Thus, it has become inevitable to complement traditional wildlife management methods with technology and artificial intelligence [8,9,10].

Asiatic lions presently range extensively in multi-use land matrices in varying administrative and legal land-use areas [5,11]. Previously, we showed that this movement has considerable intraspecific variation, with adult and sub-adult males having a greater tendency to make long-distance movements [11]. Our study established the fact that the movement of this nature is important for fulfilling life-history requirements and, therefore, crucial for the continued survival of lions [11,12]. Here, we took into account the fact that this ranging behaviour makes lions vulnerable to accidents on linear infrastructures and increases the risk of their falling into open wells, accidental electrocution and other causes of unnatural mortality [13]. We foresee that managers need to heed two categories of alerts while remotely monitoring them—one is when a lion is not moving (immobility) and the other is when it is traversing through risk-prone areas (movement). Our study demonstrated how these dual signals can be interpreted and managed using the Asiatic lion as a case study. Our renewed conservation strategy was implemented by deploying GPS radio-collars on lions, so that individual lions could be remotely monitored, almost in real time, and prompt management responses to threat situations could be triggered, thereby strengthening on-ground protection measures.

Our pilot study was conducted on 19 lions that were monitored uninterrupted for a period of one year from 2019 to 2020. This study was part of a long-term study that is being conducted in the Asiatic Lion Landscape, Gujarat, India. The paper summarizes preliminary results and provides protocols for future monitoring. The study specifically including the following: (1) exploring lion immobility and the relationship of GPS cluster points to see if a predictive model/algorithm for threats (injury) can be defined and distinguished from non-threat clusters (kill); (2) discussing on-ground action for risk alerts when lions are moving and the efficacy of the virtual geofence as an early warning to prevent accidents involving lions; (3) presenting a case study of a nomadic sub-adult lion foraying towards urban townships and the capabilities provided by GPS telemetry in ensuring the safety of the dispersing lions.

## 2. Materials and Methods

### 2.1. Study Area

**Figure 1:**
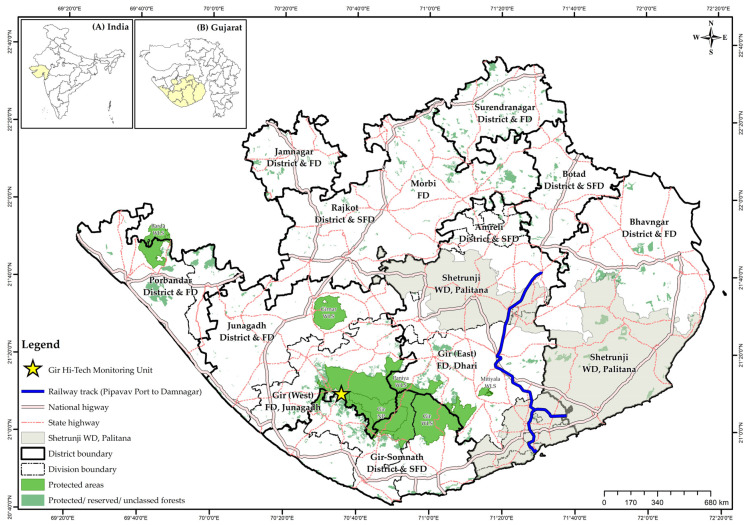
Asiatic Lion Landscape, Gujarat, India, with details of district boundaries, forest administrative boundaries, the Gir-Hi-Tech Monitoring Unit, Sasan-Gir, National and State Highways and Pipavav-Damnagar railway track. Insets in the map show Gujarat in India (**A**) and Asiatic Lion Landscape in Gujarat (**B**).

The Asiatic lions are present in Gir National Park and Wildlife Sanctuary and its surroundings, viz. Girnar Wildlife Sanctuary, Mitiyala Wildlife Sanctuary, Pania Wildlife Sanctuary, Coastal areas, Savarkundla-Liliya, and adjoining areas of Amreli and Bhavnagar districts as satellite populations. The lions moved to forested patches through conducive corridors. They are now distributed in nine districts (Junagadh, Gir Somnath, Amreli, Bhavnagar, Botad, Porbandar, Jamnagar, Rajkot and Surendranagar) of the Saurashtra region covering an expanse of ~30,000 km^2^ (permanent distribution range: 16,000 km^2^; visitation record: 14,000 km^2^), which is termed as the Asiatic Lion Landscape (Figure 1). The landscape includes five protected areas (Gir National Park, Gir Wildlife Sanctuary, Paniya Wildlife Sanctuary, Mitiyala Wildlife Sanctuary and Girnar Wildlife Sanctuary) and other forest classes, making it a total of 2058 km^2^ (1879.13 km^2^ Gir PAs + 178.87 km^2^ Girnar Wildlife Sanctuary). The Gir protected area (PA) represents India’s unique semi-arid biogeographic zone. The natural forest patches consist of dry deciduous forests and thorn forests predominantly consisting of *Tectona grandis*, *Acacia catechu*, *Terminalia crenulata*, *Diospyros melanoxylon*, *Acacia nilotica*, *Phyllanthus emblica*, *Lannea coromandelica*, *Anogeissus latifolia*, *Sterculia urens*, *Terminalia bellirica*, *Zizyphus mauritiana*, *Acacia senegal*, *Acacia leucophloea*, *Butea monosperma*, *Bauhinia racemosa*, *Wrightia tinctoria*, *Aegle marmelos*, *Cassia fistula*, etc.

Many large and small protected, reserved and unclassed forests occur around the sanctuaries. The Gir National Park and Wildlife Sanctuary and Paniya Wildlife Sanctuary serve as the primary areas for the lions and while seven other areas—Mitiyala and Girnar Wildlife Sanctuaries, South-western coast (Sutrapada-Kodinar-Una-Veraval), South-eastern coast (Rajula-Jafrabad-Nageshree), Savarkundla-Liliya and the adjoining areas of Amreli, Bhavnagar mainland and Bhavnagar coast harbour satellite lion populations. This landscape is under the jurisdiction of 13 different forest divisions [14].

### 2.2. Methodology

For radio-collaring, priority was given to individual lions that frequently moved in/near conflict areas, linear infrastructure, revenue areas, corridors, riverine areas, coastal areas, wastelands and grasslands (locally known as vidis) and on the forest fringe. Vectronic GPS satellite radio-collars (model: GPS-Plus; Vectronic Aerospace GmbH, Berlin, Germany) were deployed on these lions. The collaring exercise was carried out under the supervision of qualified and experienced wildlife veterinarians, following the required permission from the Ministry of Environment, Forests and Climate Change (MoEF&CC), the Government of India. A team of qualified, experienced wildlife veterinarians and healthcare personnel followed all safety protocols and standard operating procedures while deploying the collars. The radio-collars were configured to record GPS fixes every one hour and send the data every four-hour interval through the server on an almost real-time basis. The data were downloaded using the GPS Plus X software (Vectronic Aerospace GmbH, Berlin, Germany). These activities were coordinated from the Gir Hi-Tech Monitoring Unit, Sasan-Gir, located in the western part of Gir PA. Risk alerts for lions were also relayed from here to the concerned field staff directly or via the forest division/range administrative heads linked through an extensive wireless and/or GSM network communication system.

The present study reports data from monitoring 19 individual lions that were categorized in different age-sex categories as—small cubs (<1 year), large cubs (1 to 2 years), sub-adults (2 to 4 years), prime young (4 to 7 years), prime old (7 to 9 years) and old (>9 years) [15,16]. The radio-collars also featured a three-axis accelerometer-based activity sensor for activity data, and these data from 19 radio-collars were further analysed using activity pattern software (version 1.2.3; Vectronic Aerospace GmbH, Berlin, Germany). The activity was programmed to be logged every 5 min and was stored onboard and retrieved upon the removal/replacement of the collar.

We used ArcMap (version 10.8.1; ESRI, Redlands, CA, USA) for spatial analysis using the GIS platform. The ‘Points to Line’ tool was used for calculating the sub-adult lion’s dispersal distance. The mean (±SE) activity per day was calculated using the ‘Statistical Chart by date’ option of the software and summarised the average diurnal activity for all lions. One-hundred percentage minimum convex polygon (MCP) was used for reporting the sub-adult lions’ home range, such that every location of the lion within its range was important for management action and monitoring (the sub-adult lions were not included in this analysis).

#### 2.2.1. Immobility and Alerts

Localized movement (immobility) has been defined as clusters and is likely to be related to either kill sites or injury/ill-health [17,18,19]. The study defined clusters as ≥2 locations within 500 m of each other for a minimum of 2 days based on feeding times around kill sites of a large-sized prey [19]. To verify overlaps over feeding, the clusters were matched with livestock kill records based on criteria over space (GPS fixes of clusters within a 5 km buffer) and time (date overlaps).

The individual animal’s health status was verified at the Gir High-Tech Monitoring Unit by relaying health alerts to field staff in real time based on the criteria of localised movement (<500 m) for over 48 h. The health status of the individual lion was reported back, and if required, minimalistic wildlife veterinary care was provided to the individual. A health alert was likely to be a kill cluster but was not systematically documented by us as the kill data were not verified in real time.

Individual spatial layers for kill location fixes were created, and veterinary care records for adult lions overlaid with clusters for the monitored lions for one year between 2019 and 2020. The relationship or characteristics of clusters with lion activity was analysed and was related to the probability of a binary response (kill = 1, non-kill = 0; confirmed injury/illness = 1, not injured/ill = 0) using a subset of equal non-kill/non-injury cluster points when a kill or injury did not occur. The package mgcv [20] was used for generalized additive models (GAMs) to test the relation between activity and the binomial responses for kill and injury [19].

#### 2.2.2. Movement and Alerts

The risk of accidents, namely, collision with speeding vehicles on road and with trains on rail tracks, is related to the movement of lions. In recent years, accidents of lions have happened on the freight transportation railway track from Port Pipavav to Damnagar (Figure 1). The efficacy of geofence (linear fencing 1 km on either side of the rail track created spatially on the GIS platform) alerts for monitoring radio-collared individuals approaching or moving close to the rail tracks.

**Figure 2:**
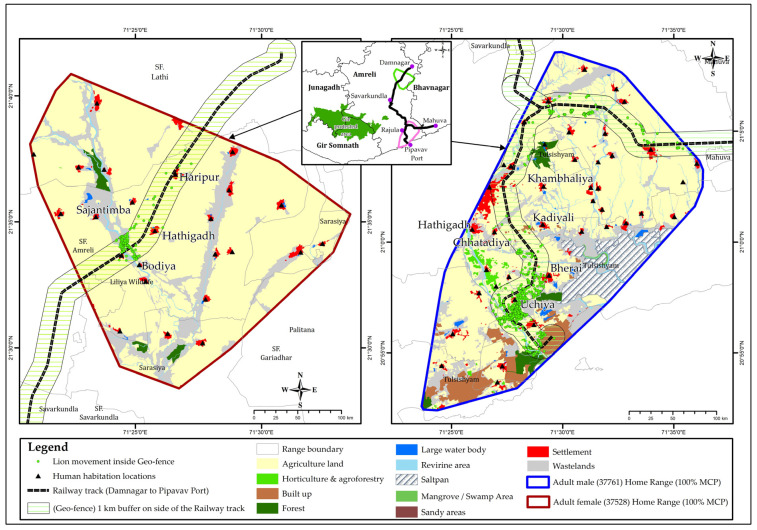
The home range of Asiatic lions, exclusively ranging in multi-use landscapes outside Gir Protected Areas (PAs), is vulnerable to linear infrastructures-related accidents such as roads and railway tracks. The safety of adult males (37761) and adult females (37528) was monitored through radio-telemetries’ virtual geofencing function.

Dispersing lions follow an unpredictable movement trajectory, such that it is challenging to keep track of their movements on the ground [11,12]. The sub-adult lion (referred to as Chotila male from here onwards) was part of a two-male coalition. The Chotila male’s natal range was in Girnar Wildlife Sanctuary (confirmed by a microchip database dated in December 2018) and was located in Surendranagar Forest Division on 17 November 2019 at a distance of 127.3 km. After two weeks of tracking, the Chotila male was deployed with a radio-collar on 3 December 2019. The collar was replaced on 27 November 2020. The dispersal of the sub-adult males was reported, until they established territory and their ranges stabilised (Figure 2).

## 3. Results

Each of the 19 radio-collared individuals was monitored for a mean (±SD) of 436.5 ± 32 days. The overall mean (±SE) diurnal activity was 20.4 ± 0.12 (range: 0–76.9; *n* = 8483).

### 3.1. Immobility and Alerts

About 269 clusters with an average of about 14.2 (±5.2) per individual for the monitoring period (Table 1) were identified. A total of 5000 kill records from all the forest divisions were explicitly analysed for spatial and temporal overlaps with intensively monitored individual lions. A total of 17% of probable kill events (*n* = 120) were found to overlap with the home ranges of the study animals, out of which only six kill events overlapped with the cluster points. A comparative analysis of movement/activity with kill and non-kill and also with injury and non-injury clusters was performed but did not yield significant results for the models tested for injury (GAM, k = 9, *p* = 0.9) or kill (GAM, k = 9, *p* = 0.71).

### 3.2. Movement

A total of 10 geofence breaches (Figure 2) for an adult female (collar ID: 37528) and 44 geofence breaches for an adult male (collar ID: 37761) occurred from August 2019 to June 2020. Ground staff followed up the alert messages with round-the-clock vigilance, until the lions exited the geofence zone of 1.0 km on either side of the railway track (Figure 2, Table 1). There was an apparent time-lapse between the GPS location, satellite data retrieval, and alert transmission, but it was yet effective for monitoring risk to the individual lion.

**Figure 3:**
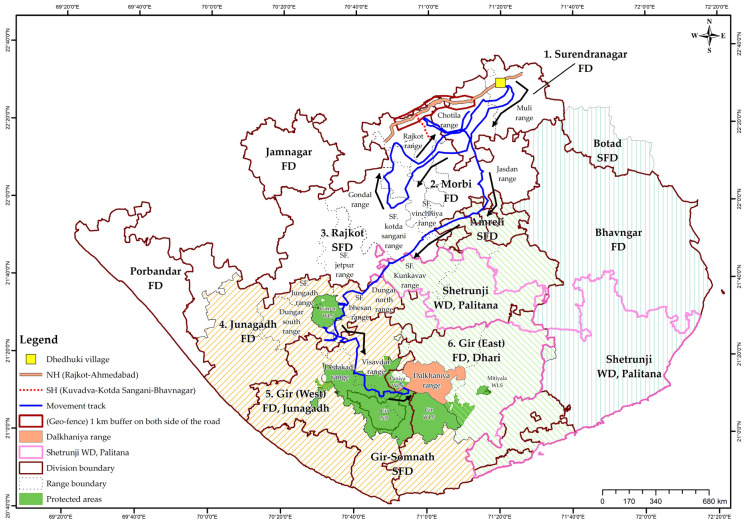
Dispersal paths of radio-collared sub-adult Chotila males and coalition partners monitored from December 2019 to June 2021. We traced movement paths from the early random forays after dispersal from natal territory till the time of stabilisation of range. SFD, social forestry division; FD, forest division; WD, wildlife division.

The Chotila males covered an aerial distance of 127.3 km from Girnar Wildlife Sanctuary to the initial dispersal site in Surendranagar Forest Division and subsequent dispersal to Gir (East) Division (Figure 3). The males stayed a maximum of two days in a single site in less than five instances during the entire nomadic phase. The large distance forays included Dheduki Village, Surendranagar Forest Division to Aji Dam, Rajkot Social Forestry Division (55 km); Aji Dam, Rajkot Social Forestry Division to Chotila Range, Surendranagar Forest Division (73 km); Jasdan Range to Dungar North Range, Junagadh Forest Division (150 km); Dungar North Range, Junagadh Forest Division to Dalkaniya Range, Gir (East) Division (89 km). The actual aerial distance from the natal range (Dungar North Range, Girnar Wildlife Sanctuary, Junagadh Forest Division) to the adult territory range [Dalkhaniya Range to Gir (East) Division] is 42 km.

**Figure 4:**
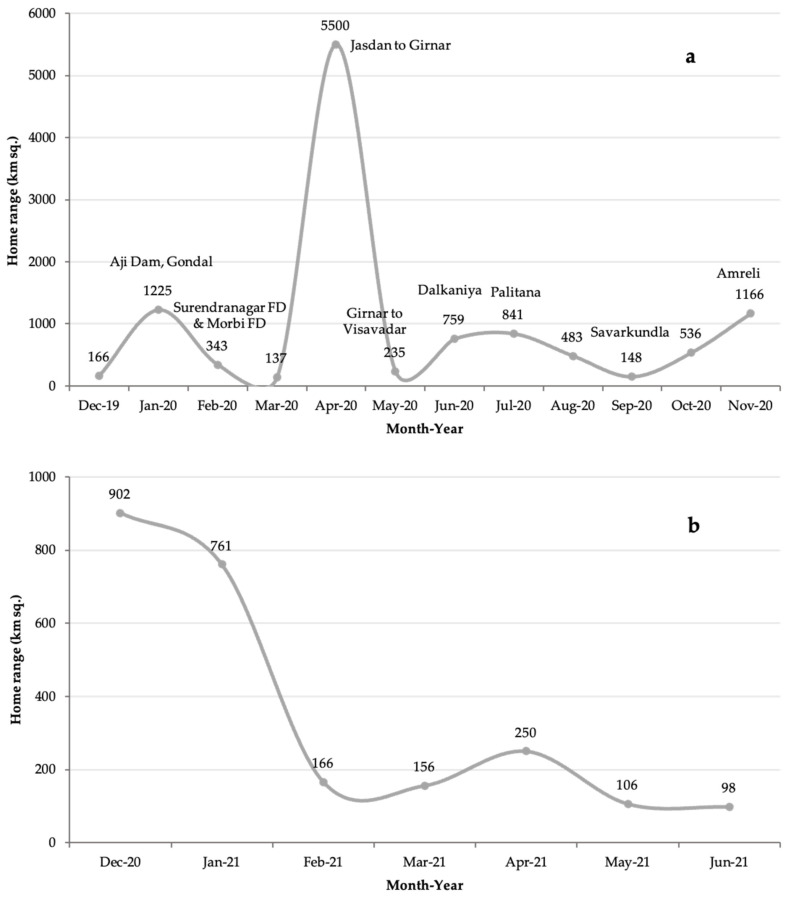
The home range (100% minimum complex polygon) of sub-adult Chotila males and coalition partners. (**a**) Home range during the nomadic stage from December 2019 to 20 November 2020; (**b**) home range during the post-nomadic phase from December 2020 to June 2021.

The home range (100% MCP) (Figure 4) during the nomadic stage from December 2019 to 20 November 2020 was 10,711 km^2^ across various districts and forest administrative units (Figure 4a). We considered the period from December 2020 to June 2021 as the post-nomadic phase based on the progressively compact nature of the home range (Figure 4b). During this phase, the home range (100% MCP) was 1480 km^2^.

From December 2019 to March 2020, the lions predated on livestock (*n* = 29), constituting buffalo (37%), cattle (52%), oxen (7%) and goat (3%), out of which 79% consisted of buffalo and cattle calves. They moved through natural grassland patches called vidis and several small and large forest patches such as Girnar Wildlife Sanctuary and Paniya Wildlife Sanctuary (Figure 1), where wild ungulates such as spotted deer (*Axis axis*), blue bull (*Boselaphus tragocamelus*) and wild pig (*Sus scrofa*) are found in good numbers [21]. However, the wild kills made by the sub-adult lions were not recorded and, therefore, not included in this study.

## 4. Discussion

**Figure 5:**
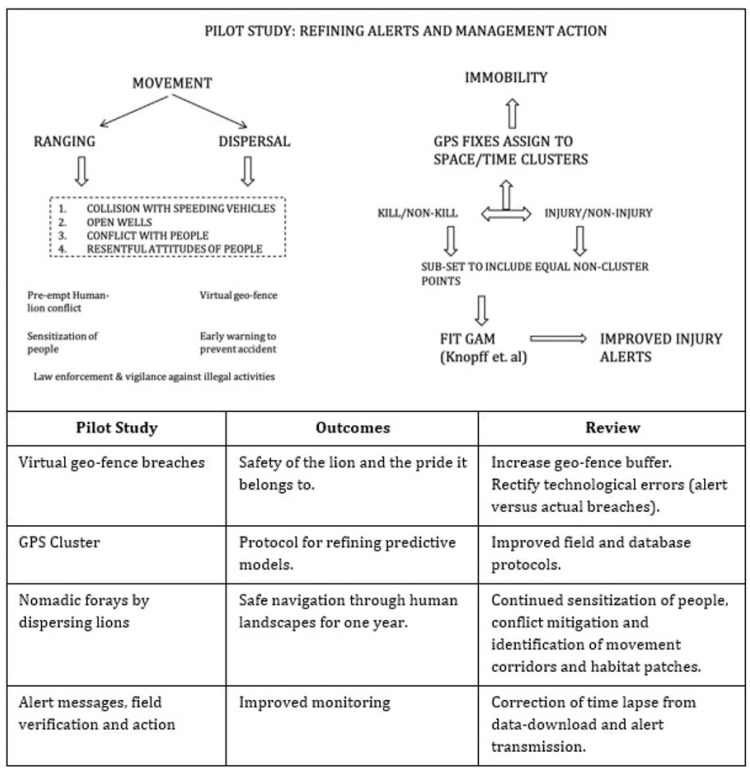
Outline of the pilot study highlighting the application of real-time spatio-temporal data for management action to conserve endangered Asiatic lions.

Radio-telemetry has greatly improved our understanding of animal space-use, habitat requirements and resource use [22]. This has been applied for over three decades to Indian wildlife, providing great ecological insights [23]. Our study presents the application of this knowledge to conservation management. We discussed the risk and response to lion immobility and movement (Figure 5) and critically examined and discussed the findings of our experience as follows.

### 4.1. Immobility

From a systematically recorded database of the health conditions of lions, the study attempted to correlate the immobility of radio-collared lions with injury/poor health conditions requiring wildlife veterinary care intervention. Only 6% of generated alerts (*n* = 468) for 19 lions monitored were verified as actual injuries on the ground. The objective of the predictive power of the GAM model applied in the study was an attempt to improve the quality of relayed alerts in space and time to implement timely action (Figure 5). The first step of achieving this is to segregate and distinguish immobility due to feeding and one due to injury (Figure 5). For solitary leopards, the time spent (<24 h to >166 h) at kill sites depends on prey biomass [19]. For lions, the prey consumption rate is related to prey biomass as well as age-sex and group characteristics which can further vary due to anthropogenic factors [24,25]. Outside protected areas, Asiatic lions feed predominantly on blue bulls (~180 kg), wild pigs (<50 kg) and domestic livestock (~300 kg), subject to prey availability [5,26]. Immobility is expected around kill sites, only when the prey biomass is large [19]. The study, therefore, considered only livestock predation to interpret kill clusters. When an alert is relayed based on the localized movement of lions to the local field staff, their brief is to only check for injury and health status of the individual. Considering the low samples of verified kills, we recommend field protocols that would record health status and associated ecological data (feeding and mating) and group characteristics during on-ground monitoring of radio-collared individuals while also monitoring their health conditions. This would enable better interpretation of immobility and help to refine proposed protocols or algorithms.

### 4.2. Movement

We considered movement in lions as related to both ranging to meet basic ecological needs as well as dispersal related to territory shift following a nomadic phase (Figure 5) [11,12]. The movement in the former case averages around 5 km (±4 SD), with a significant variation in relation to age, sex and group characteristics and seasons in the case of Asiatic lions [11]. Sub-adult and adult females exhibit more restricted movement, while sub-adult and adult males were found to foray large distances both before and after establishing stable territories [11,27,28]. Dispersal events show considerable variability in relation to habitat and anthropogenic factors during nomadic phases [5,29]. The dispersal distance for males within Gir PA was 26 ± 7.5 km and comparable to lower dispersal distances of bush-like habitats of Kruger NP [5,30,31]. Although there was a widespread movement of Chotila males in the multi-use land matrix, the dispersal distance (aerial) from the natal territory (Girnar) to the final stabilised territory (Dalkaniya) was 42 km (Figure 3). Herein, the foraying distances (mean 20 km) were also greater for adult and sub-adult males [11]. Given that movement within a lion’s home range is important, we showed how tracking of lions was realised in real time, aided by radio-telemetry, allowing for an advanced warning followed by action as lions negotiate their way through risk-prone areas (busy highways with speeding vehicles, railway tracks, open wells, electrified fences, etc.) at sensitive human-lion interaction sites [30,32,33]. Radio-telemetry-aided tracking helped to alert ground staff to safeguard and monitor the Chotila lions’ safety for one year. In this period, the lions showed range stabilisation, as they turned from nomadic to territorial males, mainly in the Dalkaniya Range of Gir (East) Division in Gir PA (Figure 2). Thus, our management facilitated the successful recruitment of nomadic sub-adult males into adult territorial males (Figure 3).

### 4.3. Review and Rectification

Local people’s attitude and cultural tolerance mechanisms gain significance when endangered species frequent multi-use land matrices play an important role in species’ survival (lions in this case) outside protected areas [1,26,34,35]. Virtual geofences, mostly relayed directly to local communities, have been greatly effective in averting conflict situations [10,36,37]. Our study also demonstrates the effectiveness of the virtual geofence (Figure 2 and Figure 3). Considering that the key factor for lion survival in the peripheral areas of Gir PA’s interface (10 km periphery) is the human cultural tolerance prevalent over many generations, and exchanged information on lion movement may further strengthen the trust and support for lion conservation [5,34]. On the other hand, in the absence of such sentiments, early warning systems may create new threats to lion safety. Dispersal of lions to historical ranges and areas predominantly near urban areas may lead to conflict escalation and increased risk of accidents in much-altered land-use areas [5,10]. Lions were last seen in the Chotila area in the mid-19th century, before they came to be restricted to the present-day Gir Wildlife Sanctuary [38]. We anticipate that the knowledge of the lion’s presence may cause slight fear and uneasiness for the local people towards the animal. Instead, field staff were deployed to keep the lions under observation, which helped improve the efficiency and effectiveness of on-ground conservation management by making use of the alerts relayed by using the tracking and virtual geofencing functions.

The spatially created virtual geofence on the 1 km rail track was remotely monitored and then communicated with ground staff via wireless communication and/or the GSM network. On analysis of the ‘alert-ground action’ data, the need to correct for time lapses (due to the pre-set data download protocols) and improved accuracy in risk alert transmission was noted. Therefore, alert transmissions are now rectified to include a much wider geofence of 3.0 km on either side of the rail track, and the information generated is used to identify further hotspots of risk for the lions along the rail tracks (Figure 5). Alert systems were also created to prevent potential human-lion conflict situations, illegal tourism, accidents and injuries involving people and lions. We recommend that the geofence extent need to be customised as per the ground situation and species concerned.

Transboundary conservation is a collective approach to managing conservation challenges and resource use and enhancing capabilities that elevate wildlife management above inter-country political and economic differences [39]. Similar challenges occur when dispersing wild animals (in this case, lions) across political administrative boundaries (Tehsils/Talukas within districts) and forest divisions with varied working mandates (wildlife, territorial and social forestry), including various land-use types (Table 1). The application of technology in tracking lions with the aid of satellite radio-telemetry and communication networks may help to better coordinate monitoring efforts. For managers, the technology’s efficacy depends on translating the knowledge of the real-time locations of animals to effective action on the ground.

## 5. Conclusions

GPS radio-telemetry has proved an especially efficient tool for monitoring endangered species, as they range extensively in multi-use landscapes with risk implications to both people and species [8]. The pilot study reported an in-depth analysis of the efficiency of satellite telemetry technology. It evaluated its design and implementation for conservation management for other endangered species dispersing extensively outside the natural forest and protected area boundaries. Telemetry studies can also aid in configuring the vast home ranges of wandering lions that have been found to occupy new territories away from the Gir PA. Moreover, many of these lions have extensively been moving and using the ‘link habitats’ [31,40].

For landscape planning approaches, the study provides evidence that tracking movement and ranging aided by GPS radio-telemetry is useful for the following: (i) triggering management action in real-time; (ii) developing a long-term database related to ecology and environment to predict lion behaviour better; (iii) developing baseline ecological information for impact assessment studies that would determine decisions to sanction infrastructure and other developmental activities; (iv) reviewing past management practices and developing predictive models for lion ecological requirements, habitat improvement and corridor facilitation.

## Figures and Tables

**Table 1:** The summary of demographic characteristics, monitoring, and the cluster of GPS locations of the lions monitored from June 2019 to June 2020.

Sr. No.	Collar ID	Age (Years)	Sex	Group size	No. of Administrative Boundaries in the Home Range		GPS days	No. of Clusters	Injury Alerts	Geofence Transgressions
					District	Forest				
1	37528	5–9	AF	14	1	3	475	21	No	10
2	37530 *^#^	8–10	AF	9	1	1	457	14	Yes	–
3	37533 *	7–8	AF	9	1	3	380	22	No	–
4	37579	6–7	AF	5	1	2	455	5	No	–
5	37756	5–9	AF	15	1	2	437	11	No	–
6	37757	5–7	AF	5	2	4	417	20	No	–
7	37758 *	5–9	AM	4	2	4	361	12	No	–
8	37761	5–9	AM	2	1	3	398	14	Yes	44
9	37766	3–5	SAF	1	1	3	457	16	No	–
10	37770	7–9	AM	1	2	5	444	11	Yes	–
11	37772	7–8	AF	3	1	5	468	20	No	–
12	37781 *	7–8	AM	1	1	2	447	9	Yes	–
13	37784	4–5	SAM	2	2	4	451	10	No	–
14	37786	4–5	SAM	3	2	5	471	15	No	–
15	37787	4–5	SAM	2	1	2	449	18	No	–
16	37798 *	5–9	AF	5	2	3	416	13	Yes	–
17	37809	3–5	SAM	1	2	3	403	6	No	–
18	37814	5–9	AF	20	1	3	457	21	No	–
19	37818 *^#^	5–9	AF	10	1	2	450	20	Yes	–
				Total	4	8				
20	37575/40867	3–4	SAM (Chotila male)	2	5	7	572	–	–	–

Note: sub-adult male 37575 was monitored from December 2019 to June 2021; cluster—kill*/injury^#^. AF, adult female; AM, adult male; SAF, sub-adult female; SAM, sub-adult male.

## Data Availability

Most of the data related to this article have been included in the paper and will be made available on request to the corresponding author.
